# Skin autofluorescence as a marker of cardiovascular risk in children with chronic kidney disease

**DOI:** 10.1007/s00467-012-2280-z

**Published:** 2012-09-15

**Authors:** Irena Makulska, Maria Szczepańska, Dorota Drożdż, Dorota Polak-Jonkisz, Danuta Zwolińska

**Affiliations:** 1 Department of Pediatric Nephrology, Wrocław Medical University, ul. Borowska 213, Wrocław, Poland; 2 Pediatric Dialysis Unit, Zabrze, Medical University of Silesia, Katowice, Poland; 3 Dialysis Unit, Jagiellonian University Medical College, Kraków, Poland

**Keywords:** Cardiovascular risk factors, Children, Dialysis, Nephrology

## Abstract

**Background:**

We examined skin autofluorescence (sAF) in chronic kidney disease children (CKD) in relation to renal function and dialysis modality.

**Methods:**

Twenty children on hemodialysis (HD), 20 on peritoneal dialysis (PD), 36 treated conservatively, and 26 healthy subjects were enrolled into the study. In all children sAF, pulse-wave velocity indexed to height (PWV/ht), left ventricular mass index (LVMI), blood pressure (BP), serum lipid profile, phosphate (P), calcium (Ca), and homocysteine were measured.

**Results:**

sAF was significantly elevated in CKD groups vs. controls and was significantly associated with PWV/ht, LVMI, BP, P, Ca × P product and homocysteine. sAF in HD and PD groups was positively correlated with dialysis vintage, and in the predialysis group negatively correlated with glomerular filtration rate (eGFR). Multiple regression analysis showed significant association of sAF with LVMI and P in the CKD patient group, and with dialysis treatment duration and BP in dialyzed children.

**Conclusions:**

In CKD children, tissue accumulation of advanced glycation end-products (AGEs) was observed. This was aggravated as eGFR declined and was related to early cardiovascular changes and some biochemical cardiovascular disease (CVD) risk markers. sAF as a non-invasive method may be a useful tool for identification of a clinical risk factors of cardiovascular disease in CKD children.

## Introduction

Cardiovascular disturbances are identified as the most serious complications in patients with chronic kidney disease (CKD) [1]. They lead to increased morbidity and mortality as compared to the general population. In particular, the patients on dialysis experience the highest risk of death [2, 3]. It has been recently established that advanced glycation end-products (AGEs) may contribute to vascular damage in addition to classical mechanisms [4]. AGEs are formed and accumulate in response to carbonyl and oxidative stress and decreased kidney excretion of AGEs precursors [5, 6]. Data from the literature show that in CKD patients, oxidative stress is increased, even at early stages [7]. The uremic toxins and modalities of renal replacement therapy enhance oxidative stress and lead to higher formation of AGEs [8, 9]. AGEs accumulate mainly in the extracellular matrix causing the alteration of functional properties of structural molecules such as collagen, which induces arterial or cardiac damage [10]. AGEs contribute to endothelial dysfunction by increasing vascular deposition of oxidized low-density lipoproteins, reducing nitric oxide concentration, enhancing oxidative stress, and causing abundant macrophage migration [11]. The interaction between AGEs and their receptors (RAGEs) induces the synthesis of proinflammatory cytokines subsequently leading to vascular damage. RAGEs are expressed on different cells, but endothelial RAGE has been indicated as the key factor for endothelial dysfunction [12, 13]. It should be emphasized that plasma levels of AGEs do not reflect tissue accumulation and, in contrast to tissue AGEs, are not predictive for cardiovascular morbidity and diastolic function deterioration [14, 15]. Until recently, determination of tissue AGEs was exclusively based on skin biopsy examination. Since the last few years, a new noninvasive optical tool, the autofluorescence reader, has given the opportunity to measure skin autofluorescence (sAF), which has proved to be well correlated with skin AGEs accumulation [16].

sAF was found to be related to age, smoking, diabetic status, and its complications in adult patients [17]. Elevated skin autofluorescence has been documented in patients with stable coronary artery disease, acute myocardial infarction [18] and in hemodialyzed adults with increased arterial stiffness [8]. Skin AF has been shown to be a predictive factor for morbidity and mortality evaluation in diabetic patients with end stage renal disease (ESRD) [19, 20]. Recently, data presented by Hartog et al. confirmed that sAF is an independent predictor of graft loss in kidney transplant recipients [21]. To date, sAF measurements in children have not been applied as a diagnostic procedure. We hypothesize that, as in the adult population, sAF might be associated with biochemical and morphologic abnormalities related to cardiovascular status in CKD children. Therefore, we investigated, for the first time, whether sAF varies in children with CKD, including stage 5 patients. We also analyzed the association of sAF with arterial stiffness, left ventricular mass index, and other risk factors for cardiovascular disease (CVD). In CKD patients, the impact of CKD stage and duration of renal replacement therapy on sAF were examined.

## Patients and methods

This is a cross-sectional multicenter study. Seventy-six children with CKD were included in examinations. The patients were divided into three groups. Twenty-six age-matched healthy children served as controls. Clinical and demographic details of the study subjects are summarized in Table 1. The first group (PD) included 20 children on peritoneal dialysis. Night intermittent peritoneal dialysis (NIPD) was performed in 12 patients and continuous cyclic peritoneal dialysis (CCPD) in eight patients. The Home-Choice device was used (Baxter International, Inc, IL, USA). Standard dialysis solutions with 1.5 % and 2.3 % glucose concentrations and 1.25 mmol/l or 1.75 mmol/l calcium concentrations were applied. The causes of CKD in this group were: urinary tract abnormalities (6), glomerulonephritis (5), polycystic kidney disease (3), neurogenic bladder (3), hereditary nephropathy (2), and hemolytic uremic syndrome (1). Thirteen children received angiotensin-converting enzyme inhibitors (ACE-i), six received calcium channel blockers, and three received β-blockers. All the subjects received calcium-containing phosphate binders, vitamin D analogs, and erythropoietin.

**Table 1:** Clinical and biochemical characteristics of investigated chronic kidney disease (CKD) groups and controls (two-by-two comparison, *p* values are shown)

Group Parameter	PD, *n* = 20	HD, *n* = 20	Pre, *n* = 36	Controls, *n* = 26	*p* value
Age (year)	14.3 ± 2.3	15 ± 3.3	14.9 ± 3.5	14.5 ± 3.3	NS
Gender (m/w)	12/8	10/10	17/19	12/14	NS
Dialysis vintage (months)	12 ± 11	19 ± 16	–	–	*p* = 0.02
BMI (kg/m^2^)	18.7 ± 3.9	18.7 ± 3.4	20.6 ± 4.1	18.8 ± 3.9	NS
SBP (mmHg)	117 ± 11^a,c^	128 ± 13^a,b,d^	115 ± 10^a^	100 ± 9	*p* < 0.0001
DBP (mmHg)	75 ± 11^a,c^	82 ± 10^a,b,d^	71 ± 8^a^	64 ± 6	*p* < 0.0001
Creatinine (mg/dl)	6.3 ± 2.6^a,d^	7.6 ± 3.4^a,d^	2.5 ± 1.6^a,b,c^	0.68 ± 0.11	*p* < 0.0001
Total cholesterol (mg/dl)	206 ± 43^a^	174 ± 33	203 ± 100^a^	162 ± 13	*p* = 0.0001
LDL cholesterol (mg/dl)	143 ± 44^a,c,d^	92 ± 31^b^	105 ± 69^b^	84 ± 6.2	*p* < 0.0001
HDL cholesterol (mg/dl)	52 ± 15	46 ± 9	50 ± 10	49 ± 4	NS
TGL (mg/dl)	179 ± 75^a^	166 ± 59	187 ± 242^a^	86 ± 6.5	*p* < 0.0001
Ca (mg/dl)	9.71 ± 0.79^c^	9.23 ± 0.56^a,b,d^	9.69 ± 0.63^a,c^	9.99 ± 0.34	*p* = 0.0002
P (mg/dl)	5.48 ± 1.81^a,c,d^	6.32 ± 1.17^a,b,d^	4.52 ± 0.97^b,c^	4.45 ± 0.54	*p* < 0.0001
Ca x P (mg^2^/dl^2^)	53.5 ± 19.4^a,d^	58.1 ± 10^a,d^	43.6 ± 8.9^b,c^	44.3 ± 5.3	*p* < 0.0001
CRP (mg/l)	2.97 ± 0.453	3.64 ± 0.98^a,b^	3.3 ± 0.65	2.5 ± 0.15	*p* < 0.0001
iPTH (pg/ml)	206 ± 177^a,c^	444 ± 524^a,b,d^	138 ± 135^c^	30.7 ± 4	*p* < 0.0001

*PD* peritoneal dialysis, *HD* hemodialysis

^a^PD, HD, Pre vs. control group

^b^HD, Pre vs. PD

^c^PD, Pre vs. HD

^d^PD, HD vs. Pre

The second group (HD) consisted of 20 children on hemodialysis. Dialysis sessions were performed three times a week (3–5 h) with polysulfone membranes. The blood flow ranged from 120 to 250 ml/min, and dialysate flow 500 ml/min. Dialysis fluid was buffered with bicarbonate and calcium content was 1.25 or 1.5 mmol/l. All children received heparin. The causes of CKD in this group were: urinary tract abnormalities (8), glomerulonephritis (6), neurogenic bladder (4), hereditary glomerulopathy (2). Nineteen children were treated with ACEi, 11 with calcium channel blockers, 2 with β-blockers. All the patients received calcium-containing phosphate binders, vitamin D analogs, and erythropoietin.

The third group (Pre) included 36 children with 2–4 stage CKD on conservative treatment. The causes of CKD were: urinary tract malformations (22), glomerulonephritis (5), polycystic kidney disease (3), hereditary glomerulopathy (2), unknown cause (2), hemolytic uremic syndrome (1), complications after chemotherapy of cancer (1). CKD stage 2 was detected in 13 children, stage 3 in 10, and stage 4 in 13 patients. Twelve subjects were treated with ACE-i, six with angiotensin receptor blockers (ARB), four calcium channel blockers, and one child with a β-blocker.

All patients with CKD stage 2–4 received treatment with calcium-containing phosphate binders, vitamin D analogs, and ten in stage 4 received erythropoietin.

Children below the age of six were excluded from the study. None of the children suffered from diabetes. During the examination period, children had no signs of infection. Patients with recent peritonitis or line infections were excluded.

Informed consent for participation in the study was obtained from all parents, and from children over 15. The research project was approved by the Wroclaw Medical University Ethics Committee.

CKD classification was based on K/DOQI guidelines from 2002 [22]. Estimated glomerular filtration rate (GFR) was determined with the Schwartz formula [23]. In all children, laboratory tests were performed and blood pressure (BP), pulse wave velocity (PWV), sAF, and left ventricular mass (LVM) measurements were recorded. Left ventricular mass index (LVMI) was calculated.

Blood samples obtained after overnight fasting were drawn from the peripheral vein in PD patients, Pre patients and the control group, and in hemodialyzed children prior to starting an HD session. Biochemical parameters: serum creatinine, total cholesterol, HDL-cholesterol, LDL-cholesterol, triglycerides (TGL), calcium (Ca), phosphate (P) concentration and Ca × P product were measured using a multichannel analyzer KONELAB30i (THERMO, Bio Merieux, France). Intact parathormone (iPTH) was measured using IRMA kit Duo PTH (Scantibodies Laboratory Inc, CA, USA). BMI was calculated as weight in kilograms divided by height in meters squared.

Blood pressure measurements with the oscillometric device were performed according to the recommendations from the fourth Report of the Blood Pressure Control in Children Working Group [24].

Pulse wave velocity (PWV) measurements were performed in the supine position after a 10-min bed rest on the carotid and femoral arteries three times. Children from the HD group were examined on an intradialytic day and children in the PD group emptied the peritoneal cavity before measurement. PWV was assessed using a high-fidelity tonometric probe (Miller Instruments Inc, Houston TX, USA) connected with a recording device SphygmoCor (AtCor Medical Pty Ltd, Sydney, Australia) and computed with appropriate software for signal analysis (Sphygmocor software AtCor Medical Pty Ltd, Sydney, Australia), according to the previously described methodology [25]. The coefficient of variation between the results of measurements of carotid-femoral PWV, in 4-h intervals, was 4.5 %. All PWV examinations were performed by one examiner. PWV values were indexed to height and presented as index PWV/ht.

Echocardiographic measurements were performed using a Sonos 1000 device (Hewlett Packard, NY, USA) with a 3.5-MHz sonographic probe. LVM was calculated according to the formula of Devereux [26]. LVM was indexed to body height according to the de Simone formula: LVMI = LVM (g) / height (m)^2.7^ [27]. All echocardiograms were stored and analyzed by one cardiologist who was blinded to the examined children.

Skin autofluorescence was assessed using the AGE Reader device (Diagnoptics BV, Groningen, The Netherlands), as described previously by Meerwald et al. [28]. In brief, the ratio of average light intensity per nanometer in the range between 420 and 600 nm emitted by the source, divided by the average of excited light intensity per nanometer in the range between 300 and 420 nm was used as the measure of autofluorescence. The intra- and inter-day coefficient of variation for autofluorescence reader measurements was 2.7 %. sAF was expressed in arbitrary units (AU). All measurements were performed on normal skin of the lower arm (ventral site, about 5 cm distal to the antecubital space), at room temperature with the patient in a seated position.

### Statistical analysis

The results were expressed as mean values ± standard deviation when a normal distribution of variables was obtained. The differences were then compared by ANOVA test. In the case of non-normal distribution, a non-parametric Kruskal–Wallis test for median values was used. For the evaluation of the relationship between factors when normal distribution of variables was obtained, the Pearson’s correlation test was performed. The Spearman’s test was applied for data with non-normal distribution. Multiple regression analysis was performed with sAF as a dependent variable. A *p* value in the ANOVA table less than 0.05 indicated a statistically significant relationship between the variables at the 95.0 % confidence level. Statistical analyses were performed using the package STATGRAPHICS (Centurion XV v.15.2.06, StatPoint Inc, VA, USA). A *p* value less than 0.05 was considered statistically significant.

## Results

Table 1 describes the clinical and biochemical characteristics of investigated CKD groups and controls with two-by-two comparison. Examined groups were comparable for age, gender, and BMI values. The highest values of SBP and DBP were obtained in children on hemodialysis. They were significantly different as compared to children in the PD, Pre, and control groups. The mean time on PD was shorter than maintenance HD duration.

Table 2 shows the detailed results of PWV/ht, LVM, LVMI, sAF measurements, and homocysteine concentration in examined groups with two by two comparison.

**Table 2:** PWV/ht, sAF, LVM, LVMI and homocysteine values in examined groups (two-by-two comparison, *p* values are shown)

Group parameter	PD, *n* = 20	HD, *n* = 20	Pre, *n* = 36	Controls, *n* = 26	*p* value
PWV/ht (m/s)	5.51 ± 0.68^a,c^	6.07 ± 0.86^a,b,d^	5.53 ± 0.69^a,c^	4.85 ± 0.62	*p* < 0.0001
sAF (10^–2^ AU)	2.46 ± 0.72^a,d^	2.61 ± 0.57^a,d^	1.9 ± 0.46^a,b,c^	1.33 ± 0.26	*p* < 0.0001
LVM (g)	92.5 ± 21.9	106.2 ± 32.3	94.2 ± 26.7	92.3 ± 20.5	NS
LVMI (g/m^2,7^)	32.9 ± 5.5^a,c,d^	37 ± 6.4^a,b,d^	27.3 ± 6.6^b,c^	26.4 ± 5.2	*p* < 0.0001
Homocysteine (μmol/l)	14.39 ± 1.88^a,d^	15.83 ± 3.29^a,d^	11.24 ± 2.74^a^	7.64 ± 1.55	*p* < 0.0001

*PWV/ht* pulse wave velocity indexed to height, *sAF* skin autofluorescence, *LVMI* left ventricular mass index

^a^PD, HD, Pre vs. control group

^b^HD, Pre vs. PD

^c^PD, Pre vs. HD

^d^PD, HD vs. Pre

The values of PWV are expressed as the index PWV/ht. The highest value of PWV/ht was obtained in the HD group and was significantly different from other groups. PWV/ht values were also increased in PD and Pre groups as compared to controls.

sAF was significantly higher in all groups of children with CKD compared to controls. The highest absolute sAF values were observed in HD group, but we failed to demonstrate a significant difference in comparison with the PD group. LVM was not different between the examined groups. LVMI showed a significant increase in all children with CKD as compared to healthy children. The highest values were recorded in HD and were significantly different from results in PD and Pre groups. Homocysteine concentration was significantly elevated in both groups of dialyzed patients. The pre-dialysis CKD group was split into CKD2-3 and CKD4-5 stages. The sAF values in the CKD 2–3 group were mean 1.72 ± 0.41 and 2.23 ± 0.36 in the CKD 4–5 group. Differences were significant, with *p* = 0.0007.

### Analysis of factors associated with sAF

**Fig. 1:**
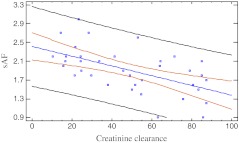
Correlation between sAF and creatinine clearance in predialysis children (*r* = –0.37; *p* = 0.0003). sAF - skin autofluorescence (10^–2^AU), creatinine clearance (ml/min/1.73 m^2^ *)*

**Fig. 2:**
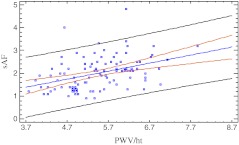
Correlation between sAF and PWV/ht in all CKD children (*r* = 0.24; *p* = 0.03). sAF - skin autofluorescence (10^*–2*^AU), PWV/ht - pulse wave velocity/height (m/s)

In dialyzed children (PD and HD), significant positive linear correlations of sAF with dialysis treatment duration, P and Ca × P were found. In children with CKD, when we analyzed all three groups (PD, HD, Pre), significant positive correlations of sAF with LVMI, PWV/ht, SBP, DBP, P, Ca × P and homocysteine concentration were revealed. A negative correlation of sAF and creatinine clearance was detected in the pre-dialysis group (*p* = 0.0003). Detailed data are shown in Table 3, and Figs. 1 and 2.

**Table 3:** Analyses of factors correlated with sAF (10^–2^ AU) (Pearson correlation test)

Variable	*r*	*p* value
sAF (10^–2^AU) (PD + HD + Pre)		
LVMI (g/m^2,7^)	0.44	0.0001
PWV/ht (m/s)	0.24	0.03
homocysteine (μmol/l)	0.26	0.02
SBP (mmHg)	0.28	0.01
DBP (mmHg)	0.23	0.03
P (mg/dl)	0.46	<0.0001
CaxP (mg^2^/dl^2^)	0.44	<0.0001
sAF (10^–2^AU) (PD + HD)		
dial. treatment duration (months)	0.46	<0.0001
P (mg/dl)	0.33	0.03
CaxP (mg^2^/dl^2^)	0.32	0.03
sAF (10^–2^AU) (Pre)		
Creatinine clearance (ml/min/1.73 m^2^)	0.42	0.0003
sAF (10^–2^AU) (Control)		
Ca (mg/dl)	0.51	0.007

*sAF* skin autofluorescence, *LVMI* left ventricular mass index, *PWV/ht* pulse wave velocity indexed to height, *SBP* systolic blood pressure, *DBP* diastolic blood pressure

In dialyzed children (PD, HD), a multivariate regression analysis revealed significant positive association of sAF with dialysis treatment duration and SBP. In the total CKD patients (PD, HD, Pre), a significant positive association of sAF with LVMI and P was found. The detailed data are shown in Table 4.

**Table 4:** Multiple regression analyses of factors associated with skin autofluorescence (sAF)

Variable	R^2^	β	95 % CI	*p* value
sAF (10^–2^AU) (PD + HD + PRE)				
LVMI (g/m^2,7^)	36.9 %	0.01	–0.003 –0.03	0.03
P (mg/dl)	36.9 %	0.06	–0.29 –0.41	0.02
sAF (10^–2^AU) (PD + HD)				
dial. treatment duration (months)	28.8 %	0.05	–0.09 to –0.01	0.02
SBP (mmHg)	28.8 %	0.08	–0.01– 0.01	0.03

*LVMI* left ventricular mass index, *SBP* systolic blood pressure, *PD* peritoneal dialysis, *HD* hemodialysis

## Discussion

This is the first study demonstrating an elevation of sAF in children with various stages of CKD and the relationship of sAF with other cardiovascular risk factors, including increased arterial stiffness and LVMI, hypertension, disturbed lipid metabolism, mineral bone disease, and high homocysteine levels. AGEs are generated as the result of protein glycation (Maillard reaction) under physiological conditions. The process is more pronounced in a hyperglycemic state and during oxidative and carbonyl stress [14]. Free circulating AGEs are cleared from the bloodstream by glomerular filtration with subsequent reabsorption in renal tubules and further degradation. A certain level of AGEs is not cleared and accumulates in tissues with low turnover [29].

Patients with stage 5 CKD are particularly at risk of accumulation of AGEs because of overproduction de novo as the consequence of increased oxidative stress. On the other hand, the clearance of AGEs precursors also decreases towards zero [30]. Our study in children also confirmed this observation. Increased values of sAF were documented in all groups of examined children with CKD as compared to healthy controls. The highest values were obtained in hemodialyzed children. However, these were not significantly different from the values observed in children on PD. Therefore, our results are consistent with those presented for adult CKD patients [7, 31]. Meerwald et al. [20] revealed two-fold higher values of sAF in 109 patients on HD as compared to healthy subjects. Moreover, they showed that sAF was an independent predictor of increased cardiovascular mortality. The causes of sAF increase are multifactorial and not only related to end-stage kidney damage but also to modality of dialysis. PD, which includes the use of glucose concentration fluids, particularly has an influence on AGEs generation. Administration of PD solutions with low GDP (glucose degradation products) content reduces plasma AGE levels and may improve cardiovascular risk profile [32]. On the other hand, bio-incompatible materials (i.e., dialysis membranes) in patients on hemodialysis aggravate oxidative stress leading to an increase of AGEs. It should be stressed that a single dialysis session is not effective for removal of AGEs, and for instance 3-deoxyglucozone is not eliminated at all [33–35]. Our multivariate analysis demonstrates that the duration of dialysis treatment is an independent predictor of increased sAF.

Significantly lower values of sAF in children with CKD treated conservatively could be explained by higher renal clearance for AGEs and by the dietary regimen with low protein content. However, also in this group, sAF values were significantly higher than in the healthy control group. This study shows that sAF gradually increases with a simultaneous decline of glomerular filtration. Recently, similar results were published in an adult CKD population [36]. It should be underlined that such an association was not found with circulating AGEs [37]. Therefore, sAF is more a suitable method for the evaluation of AGEs accumulation.

There are only a few manuscripts on pediatric populations with CKD that deal with the problem of AGEs. Sebekova et al. [38] examined plasma concentration of AGEs (fluorescent molecules, carboxy-methyl-lysine - CML) in a CKD group, which was composed of 18 children on conservative treatment, 10 on dialysis, and 9 transplanted patients. Increased AGEs levels were found in all children, with the highest values in subjects on dialysis. No significant correlation between AGEs levels and inflammatory marker concentrations (CRP, IL-6, TNF-α) was not found. These results are concordant with ours obtained during the sAF evaluation, but the interpretation should be slightly different. There is no direct correlation of sAF with circulating AGEs, but only with CML [37]. Studies on the relationship of AGEs with cardiovascular complications in patients with stage 5 CKD showed that AGEs play a role in the pathogenesis of vascular and cardiomyocyte alteration. They were localized in aortic atherosclerotic plaques and cardiomyocytes and in calcifications of coronary arteries [7, 15, 31]. Recent studies have revealed a significant correlation of plasma pentosidine and atherosclerotic lesion progression, measured by changes in the carotid intima-media complex [39]. Similar association was shown in adult patients with cardiovascular disease without CKD [20]. There are only limited data available on sAF relationship with cardiovascular risk factors in patients with CKD. These studies were conducted in subjects on maintenance dialysis. In our study the significant association of sAF and PWV/ht was shown for the children with CKD. Similar results were obtained by Ueno et al. [7] in a population of 120 adult patients on hemodialysis. They confirmed that AGEs are key factors in the pathogenesis of vascular stiffness. Vishvanath et al. [40] observed such a relationship in their study on pentosidine concentration in skin specimens from patients with type 1 diabetes. They documented a positive correlation with pulse pressure as a measure of vascular stiffness. In the Baltimore Longitudinal Study of Aging (BLSA), conducted in relatively healthy, community-dwelling adults, the results showed that serum AGEs, as represented by CML, were associated with increased arterial stiffness even after excluding all diabetic patients [4].

In our study population we did not detect LVM hypertrophy in any group, but LVMI was markedly higher in dialyzed patients as compared to healthy children and the conservative treatment group. It should be emphasized that during the two-year observation study of Mitsnefes et al., LVMI was a predictive factor for LV hypertrophy in children with CKD on conservative treatment [41]. Also Zocalli et al. documented, in a large prospective study conducted in adult patients on hemodialysis, that LVMI is an unfavorable predictor of cardiovascular events [42].

In this current study, we describe for the first time that sAF positively correlates with LVMI in a group of children with CKD. This confirms the role of AGEs in the pathogenesis of cardiac myocyte alteration. It is also possible that the impact of AGEs is indirect, mediated by their influence on vascular alteration. The positive correlation between PWV/ht and LVMI in children with CKD (*R* = 0.32; *p* = 0.003) supports this hypothesis. We discovered a close relationship of sAF with blood pressure values, which is consistent with the results of a Japanese group [43].

In this study, we analyzed, for the first time, the association of tissue AGEs with hyperhomocysteinemia, which is a known cause of endothelium damage [44, 45]. We documented that sAF shows a strong correlation with homocysteine in children with CKD. Similar association was found with classical risk factors, such as serum phosphate and calcium-phosphorus product. The influence of hyperphosphatemia, hypercalcemia, and high values of calcium-phosphorus product on the progression of vascular calcification and stiffening of vascular wall has been previously documented.

Our study has several limitations. Firstly, the sample size, particularly in subgroups, may be insufficient to show statistical differences. One should emphasize that the pediatric population with CKD is markedly smaller as compared to the population of adult patients with CKD. The epidemiological data estimate that children with stage 5 CKD account for only 2 % of the corresponding adult population [46]. Our patients were recruited from the three nephrological centers for children, which treat patients from the whole south region of our country.

Future studies with larger sample sizes are necessary, especially in the light of new therapeutic possibilities, which are responsible for the lowering of AGEs. These animal experiments have documented that some AGE cross-link breakers may prevent alterations in vessels and heart myocytes [47, 48]. Lewis et al. suggest that patients with less-advanced stages of CKD might benefit received pyridoxamine that might inhibit the formation of AGEs [49].

In summary, our study showed for the first time that in children with CKD, sAF, as a measure of tissue AGEs accumulation, is elevated simultaneously with GFR decline. The highest values of sAF were detected in patients on maintenance dialysis, probably due to further increases of oxidative stress related to the dialysis procedure. As sAF was associated with early cardiovascular changes and some biochemical CVD risk factors, our study supports the clinical association of AGEs with development of cardiovascular complications. Therefore, sAF as a non-invasive method may be a useful tool for assessment of CVD risk factors in children with CKD.
